# TALK-1 and TALK-2 channels in pancreatic β-cell function

**DOI:** 10.3389/fphar.2026.2015446

**Published:** 2026-10-30

**Authors:** Anjas Happy Prayoga, Dawon Kang

**Affiliations:** 1 Department of Physiology, College of Medicine, Gyeongsang National University, Jinju, Republic of Korea; 2 Department of Convergence Medical Science, Gyeongsang National University, Jinju, Republic of Korea; 3 Institute of Medical Science, Gyeongsang National University, Jinju, Republic of Korea

**Keywords:** calcium, diabetes, insulin secretion, KCNK16, KCNK17, TALK-1, TALK-2

## Abstract

Diabetes is a major non-communicable disease in which impaired insulin secretion contributes to loss of glucose homeostasis. ATP-sensitive K^+^ (K_ATP_) channels couple glucose metabolism to β-cell electrical activity and are established therapeutic targets. However, K_ATP_-dependent depolarization alone does not fully explain the regulation of insulin secretion. Emerging evidence identifies the two-pore-domain K^+^ channels TALK-1 and TALK-2 as additional regulators of β-cell function. This Mini Review integrates genetic, mechanistic, and pharmacological evidence to examine how TALK channels regulate β-cell function and to distinguish established cellular effects from unresolved implications for diabetes. TALK-1 studies link membrane excitability, endoplasmic reticulum (ER) Ca^2+^ homeostasis, and mitochondrial function to secretory regulation, while disease-associated variants support its relevance to diabetes. Recent human islet experiments show that TALK-2 regulates ER Ca^2+^ handling and modulates basal insulin release and the secretory response to glucose. Together, these findings indicate that the secretory consequences of TALK channel modulation depend on glucose availability and metabolic context. Selective TALK-1 inhibitors provide tools to test mechanisms previously investigated mainly through genetic manipulation. Key unresolved questions concern the relative contributions of plasma membrane and ER channels, the effects of sustained inhibition, and consequences for other islet hormones. We discuss how addressing these questions can establish whether TALK channel modulation improves glucose-responsive insulin secretion and preserves islet function, and whether these effects translate into sustained improvements in glucose homeostasis *in vivo*.

## Introduction

Non-communicable disease (NCD), including diabetes, accounted for at least 43 million deaths worldwide in 2021 (World Health Organization, 2025). An estimated 830 million people were living with diabetes in 2022 (World Health Organization, 2024). Maintaining glycemic control remains challenging, as illustrated by the Glycemia Reduction Approaches in Type 2 Diabetes (GRADE) trial, in which glycemic deterioration was common over a mean follow-up of 5 years despite the addition of glucose-lowering therapies to metformin in people with type 2 diabetes (Group et al., 2022). These challenges motivate further investigation of the cellular mechanisms governing β-cell function.

Potassium (K^+^) channels help determine pancreatic β-cell membrane potential and regulate the Ca^2+^ influx that triggers insulin secretion (Rorsman and Ashcroft, 2018). Their clinical relevance is exemplified by ATP-sensitive K^+^ (K_ATP_) channels: gain-of-function mutations in *KCNJ11*, encoding Kir6.2, can cause neonatal diabetes (Gloyn et al., 2004), and sulfonylurea treatment can enable affected patients to discontinue insulin while improving glycemic control (Pearson et al., 2006). Although this example establishes the value of targeting a specific channel defect, the contribution and therapeutic relevance of other β-cell K^+^ channels require independent investigation.

TALK-1 (K_2P_16.1, encoded by *KCNK16*) and TALK-2 (K_2P_17.1, encoded by *KCNK17*) are members of the two-pore-domain potassium (K_2P_) channel family that extend the regulation of β-cell function beyond K_ATP_-dependent initiation of electrical activity. TALK-1 modulates glucose-stimulated electrical activity and endoplasmic reticulum (ER) Ca^2+^ homeostasis (Vierra et al., 2015; Vierra et al., 2017). Human islet studies further demonstrated that TALK-2 regulates ER Ca^2+^ handling and influences both basal insulin release and the secretory response to glucose (Dobson et al., 2026). These findings connect TALK channel function to membrane excitability and intracellular Ca^2+^ regulation, highlighting the importance of glucose conditions and subcellular localization when interpreting the effects of channel modulation.

The relevance of these mechanisms to diabetes differs across disease forms. The rare KCNK16 p. L114P gain-of-function variant was identified in a family with maturity-onset diabetes of the young (MODY), and functional studies linked increased TALK-1 activity to impaired insulin secretion (Graff et al., 2021). Common susceptibility variants involving KCNK16 and regulatory variation associated with KCNK17 expression instead implicate TALK channels in type 2 diabetes risk (Vierra et al., 2015; Dobson et al., 2026). These findings support mechanistic investigation in monogenic secretory dysfunction and type 2 diabetes, but do not establish therapeutic efficacy in either setting. Their relevance to type 1 diabetes requires separate evaluation and cannot be inferred from genetic associations or studies of insulin secretion alone.

Previous reviews have discussed TALK channels within broader accounts of islet electrophysiology, ER Ca^2+^ dysregulation, and diabetes (Rorsman and Ashcroft, 2018; Jacobson and Shyng, 2020; Yang and Yang, 2025; Dobson and Jacobson, 2024), while a recent review of β-cell mitochondrial homeostasis also considers TALK-1-mediated intracellular Ca^2+^ regulation (Li et al., 2026). Human TALK-2 functional studies and the development of selective TALK-1 inhibitors now provide additional evidence for a focused assessment of these channels (Dobson et al., 2026; Nakhe et al., 2026). Here, we examine how TALK-1 and TALK-2 biophysical properties and subcellular localization shape β-cell Ca^2+^ handling and insulin secretion. We integrate genetic and pharmacological findings while distinguishing cellular effects from changes in whole-animal glucose homeostasis, and identify the evidence needed to determine whether TALK modulation can produce sustained improvements in β-cell function.

### Expression and biophysical properties of TALK channels

TALK-1 and TALK-2 are alkaline-activated members of K_2P_ channel family initially identified through their prominent expression in the human pancreas (Girard et al., 2001). An early *in situ* hybridization study detected both transcripts in acinar cells but not in pancreatic islets (D et al., 2005). Subsequent studies established functional TALK-1 expression in mouse and human β-cells and TALK-2 expression and function in human β-cells (Vierra et al., 2015; Dobson et al., 2026). This distinction is important because *KCNK17* is absent from the rodent genome, limiting the use of conventional rodent models to investigate TALK-2 physiology (Dobson et al., 2026). In transfected COS-7 cells, TALK-1 and TALK-2 exhibited single-channel conductances of approximately 21 and 33 pS, respectively, at −60 mV in symmetrical 150 mM KCl. Extracellular pH modulated TALK-1 activity over pH 6–10, whereas TALK-2 responded predominantly over pH 7–10; changes in activity mainly reflected altered opening frequency (Kang and Kim, 2004). These measurements define channel behavior under specified recording conditions, rather than the magnitude of native β-cell currents. Mechanistically, changes in channel opening can alter the background K^+^ conductance that opposes depolarization and thereby influence Ca^2+^ entry and insulin secretion. However, whether physiological pH changes regulate secretion through TALK channels in intact islets remains to be established.

Subunit composition further modifies this relationship. TALK-1 can assemble with TALK-2 to form functional heterodimers, and association between endogenous subunits has been detected in human EndoC-βH5 cells. Heteromerization shifts TALK-2-containing channels toward responsiveness to extracellular alkalinization within the physiological range (Khoubza et al., 2022). Thus, homomeric channel properties may not fully predict native TALK activity. This provides a possible link between local pH, channel assembly and secretory regulation, although the contributions of defined heteromers to β-cell electrical activity, ER Ca^2+^ handling and insulin secretion remain unresolved.

### Electrical and intracellular control of insulin secretion

At the β-cell plasma membrane, TALK-1 contributes to the K^+^ conductance that persists as glucose metabolism reduces K_ATP_ channel activity and initiates depolarization. In mouse islets, TALK-1 deletion increased electrical excitability, Ca^2+^ influx, and second-phase glucose-stimulated insulin secretion (GSIS) (Vierra et al., 2015). Thus, TALK-1 helps shape the electrical activity and Ca^2+^-dependent secretory response that follow the initial glucose-induced depolarization.

TALK-1 also influences intracellular Ca^2+^ handling through its activity in the ER. Nuclear-envelope recordings and Ca^2+^ measurements support a model in which ER-localized TALK-1 provides a K^+^ countercurrent that facilitates Ca^2+^ release. Compensating K^+^ movement limits the charge imbalance generated as Ca^2+^ leaves the ER, allowing continued release without TALK-1 itself conducting Ca^2+^. Loss of TALK-1 reduced ER Ca^2+^ leak and increased ER Ca^2+^ stores; islets from knockout mice also showed reduced expression of ER stress-associated genes after prolonged high-fat feeding in mice (Vierra et al., 2017).

Human β-cell studies identify a related intracellular role for TALK-2. Knockdown reduced ER TALK-2-like channel activity and increased ER Ca^2+^ storage. In human pseudoislets, β-cell-specific knockdown lowered basal insulin release and increased the glucose-stimulated secretory response relative to baseline, whereas whole-cell K_2P_ currents were not significantly altered under the tested conditions (Dobson et al., 2026). These findings support a contribution of ER-localized TALK-2 to the regulation of secretion, although the steps linking altered ER Ca^2+^ handling to the distinct basal and glucose-stimulated responses remain to be fully resolved.

### Genetic evidence connecting TALK channels to diabetes

The strongest direct human genetic evidence concerns the *KCNK16* p. L114P gain-of-function variant identified in a family with MODY (Graff et al., 2021). Functional studies showed increased TALK-1 conductance, impaired glucose-induced depolarization and Ca^2+^ entry, reduced ER Ca^2+^ storage, and diminished insulin secretion in experimental islet preparations (Graff et al., 2021). Mice carrying the corresponding variant developed neonatal hyperglycemia and adult glucose dysregulation, with severity influenced by genotype and genetic background (Nakhe et al., 2024). Together, these findings support a mechanism in which excessive TALK-1 activity disrupts glucose-stimulated insulin secretion. Increased glucagon secretion in the knock-in model also suggests that altered islet hormone balance contributes to the phenotype (Nakhe et al., 2024).

Common susceptibility variants provide a link to type 2 diabetes, although their causal interpretation is less direct. The risk-associated variant rs1535500 encodes TALK-1 A277E, which increases channel open probability and surface expression (Vierra et al., 2015). These functional effects suggest how the variant could impair insulin secretion, but do not establish TALK-1 as the sole mediator of risk at the locus. Colocalization of type 2 diabetes genome-wide association study (GWAS) signals with human islet expression quantitative trait loci also implicated *KCNK17* regulation, prioritizing rs34247110 as a candidate variant whose risk allele is associated with increased TALK-2 expression (Dobson et al., 2026). Human TALK-2 knockdown experiments provide complementary evidence that altered channel expression influences insulin secretion, but do not establish that the candidate risk allele acts through this mechanism. Variant-specific perturbation is needed to test that causal relationship.

These findings distinguish a rare coding variant with functional and *in vivo* support for pathogenicity from common susceptibility signals whose contributions must be resolved within a polygenic background. They justify investigating TALK-dependent secretory dysfunction in defined genetic contexts, but do not by themselves predict the response to pharmacological inhibition.

### Metabolic stress and islet hormone regulation

The contribution of TALK channels to insulin secretion also depends on the metabolic state of the β-cell. β-cell-specific TALK-1 deletion increased mitochondrial Ca^2+^ accumulation and ATP production, linking the channel’s effects on cellular Ca^2+^ handling to metabolic coupling. Under high-fat feeding, β-cell-specific TALK-1 knockout mice showed improved glucose tolerance despite lower fasting and glucose-stimulated circulating insulin levels; isolated islets from these mice also exhibited lower GSIS than those from diet-matched controls (Graff et al., 2024). Thus, the relevance of TALK-1 extends beyond an acute increase in GSIS to the adaptation of β-cell function during sustained metabolic demand. These mitochondrial effects do not by themselves establish a functional TALK-1 channel in the mitochondrial membrane.

Inflammatory studies further illustrate the context dependence of TALK-1 function. Low-dose cytokine exposure reduced TALK-1 expression and activity, increased ER Ca^2+^ storage, and helped maintain second-phase glucose-stimulated Ca^2+^ influx and GSIS. In TALK-1-deficient mouse islets, this adaptive response was absent, and cytokine treatment reduced second-phase Ca^2+^ influx and GSIS relative to untreated knockout islets (Dickerson et al., 2018). These findings indicate that stress-induced downregulation of TALK-1 and constitutive channel deletion are not functionally equivalent. They describe adaptation to low-grade inflammatory stress and do not establish a therapeutic benefit of TALK-1 inhibition in autoimmune type 1 diabetes. TALK-1 function is also regulated through protein interactions. Intracellular osteopontin increased channel activity in a manner dependent on the TALK-1 splice variant and promoted membrane hyperpolarization in primary mouse β-cells (Dickerson et al., 2017). Thus, changes in channel abundance and regulation of channel activity represent distinct influences on β-cell function. Assessing TALK-1 expression together with its activity and downstream Ca^2+^ responses is therefore important for understanding its contribution under metabolic and inflammatory stress.

The intact-islet response adds a further layer of regulation. In δ-cells, TALK-1 limits ER and cytoplasmic Ca^2+^ signals and somatostatin secretion. TALK-1 deletion increased somatostatin release and reduced glucagon secretion, with restoration of glucagon secretion after blockade of somatostatin receptor signaling (Vierra et al., 2018). Consequently, an islet-wide inhibitor may affect insulin secretion both directly through β-cells and indirectly through paracrine interactions. Measuring insulin, glucagon and somatostatin together is therefore necessary to interpret its net effect on islet function.

Whole-animal findings also remain model dependent. A recent constitutive *Kcnk16* knockout study reported largely preserved glucose homeostasis despite reduced growth and adiposity and modest changes in insulin secretion dynamics (Früh et al., 2026). This differs from the protection against diet-induced glucose intolerance observed after β-cell-specific deletion (Graff et al., 2024). Differences in targeting, developmental adaptation and experimental conditions are possible explanations, but have not been resolved. Comparisons of pharmacological inhibition, inducible deletion and constitutive knockout under matched conditions would clarify which effects reflect reversible channel modulation and which depend on lifelong gene loss.

The integrated roles of TALK-1 and TALK-2 in β-cell excitability, Ca^2+^ homeostasis, and insulin secretion, together with pharmacological modulation of TALK-1, are summarized in Figure 1.

**FIGURE 1:**
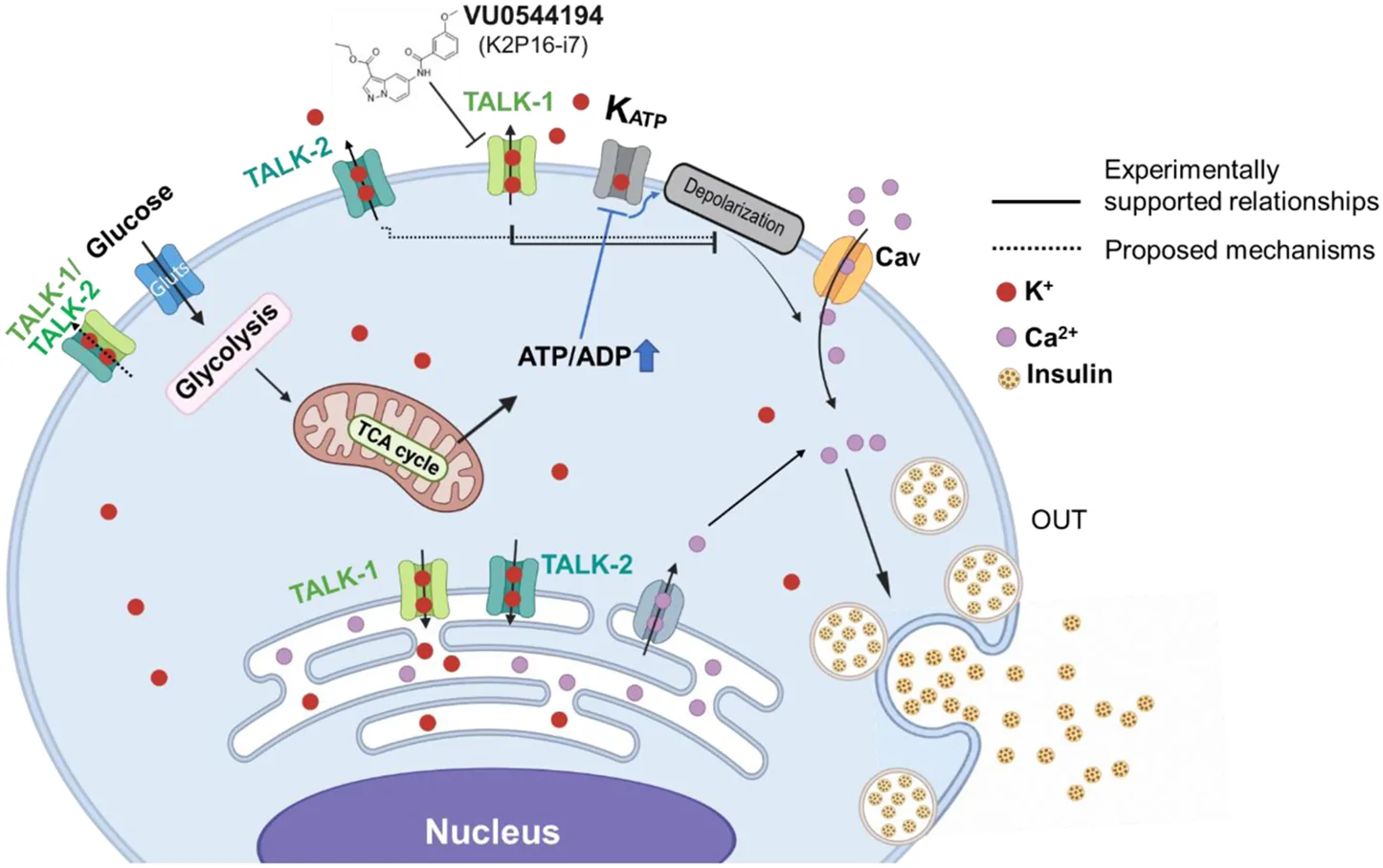
Roles of TALK-1 and TALK-2 channels in pancreatic β-cell Ca^2+^ homeostasis and insulin secretion. Glucose uptake and metabolism increase the intracellular ATP/ADP ratio, promoting ATP-sensitive K^+^ (K_ATP_) channel closure, membrane depolarization, and opening of voltage-dependent Ca^2+^ channels (VDCCs). The resulting Ca^2+^ influx triggers insulin granule exocytosis. Plasma membrane TALK-1 (K_2P_16.1, encoded by *KCNK16*) provides a background K^+^ conductance that counteracts depolarization and limits glucose-stimulated Ca^2+^ entry and insulin secretion. TALK-2 (K_2P_17.1, encoded by *KCNK17*) and TALK-1/TALK-2 heteromers are also depicted; their specific contributions to plasma membrane excitability in native β-cells remain to be defined. At the endoplasmic reticulum (ER), functional studies support a model in which TALK-mediated K^+^ countercurrents facilitate Ca^2+^ leak or release through separate Ca^2+^-permeable pathways, thereby influencing ER Ca^2+^ storage and cytosolic Ca^2+^ signaling. TALK channels do not themselves conduct Ca^2+^. In human β-cells and pseudoislets, TALK-2 knockdown increased ER Ca^2+^ storage, reduced basal insulin release, and enhanced the glucose-stimulated secretory response relative to baseline. The mechanisms connecting these ER changes to glucose-dependent Ca^2+^ entry and secretion remain incompletely resolved. The TALK-1 inhibitor VU0544194 (K2P16-i7) reduced β-cell K_2P_ currents and enhanced glucose-stimulated Ca^2+^ signaling and insulin secretion in mouse preparations. A separate assay in TALK-1-expressing heterologous cells supported an increase in releasable ER Ca^2+^ stores, without directly establishing inhibition of ER-localized TALK-1 currents. Solid connections indicate experimentally supported relationships, whereas dotted connections indicate unresolved contributions in native β-cells. These distinctions concern the indicated relationships rather than the presence or absence of channel function. The schematic integrates evidence from heterologous expression systems, mouse preparations, and human β-cells or islets. Red and purple circles represent K^+^ and Ca^2+^, respectively; gold particles represent released insulin. Arrowheads indicate ion movement or stimulation, and blunt-ended lines indicate inhibition.

### Pharmacological evidence and its limitations

VU0544194 (K2P16-i7) provides a pharmacological tool for investigating the contribution of TALK-1 to β-cell function (Nakhe et al., 2026). Nakhe et al. identified this compound through thallium-flux screening in T-REx cells expressing human TALK-1, with an IC_50_ of 5.20 μM in the initial concentration–response assay. In mouse β-cells and islet preparations, VU0544194 reduced K_2P_ currents, increased the fraction of time spent at elevated cytosolic Ca^2+^ during glucose stimulation, and enhanced insulin secretion. During perifusion of C57BL/6J islets with a glucose increase from 2.0 to 16.7 mM, normalized insulin-secretion areas under the curve (AUC) increased 1.59-fold overall and 1.68-fold during the second phase (n = 3 mice). In a separate assay, treatment of TALK-1-expressing T-REx cells with 10 μM VU0544194 for 1.5 h increased cyclopiazonic acid-evoked Ca^2+^ mobilization, consistent with a larger releasable ER Ca^2+^ pool. This observation supports an effect on ER Ca^2+^ homeostasis but does not directly demonstrate inhibition of ER-localized TALK-1 currents. Counter-screening against Kir4.2, TALK-2, TASK-3, K_ATP_, and hERG supported the selectivity of VU0544194 for TALK-1 within the tested channel panel, although activity against additional targets remains to be characterized (Nakhe et al., 2026). The study did not evaluate the compound in human islets or assess glucose regulation *in vivo*. Thus, the acute enhancement of insulin secretion in isolated mouse islets does not establish efficacy in human β-cells or sustained glycemic benefits at tolerated exposures.

Whether selective pharmacological inhibition reproduces the effects of TALK-2 knockdown in human pseudoislets remains unresolved (Dobson et al., 2026). Furthermore, functional heteromerization of TALK-1 and TALK-2 raises the question of whether compounds characterized on homomeric channels retain their activity in native channel assemblies (Khoubza et al., 2022). TALK-1 inhibition, TALK-2 inhibition, and combined modulation should therefore be evaluated as distinct interventions.

Further pharmacological studies should establish how selective TALK modulation affects glucose-responsive secretion in human islets, including those from donors with type 2 diabetes. Measurements of channel activity, compartment-specific Ca^2+^ signals, and dynamic hormone secretion at matched compound concentrations would help connect channel inhibition to functional outcomes. Basal and stimulated insulin secretion should be assessed separately, because a higher stimulation index can result from a lower baseline without an increase in stimulated release. Parallel measurements of glucagon and somatostatin would clarify effects on coordinated islet hormone secretion. Attenuation of drug responses after genetic target depletion would strengthen attribution to the intended channel, while longer exposure should determine whether functional benefits persist without depletion of insulin stores, ER dysfunction, or loss of viability.

Subsequent *in vivo* studies should determine whether these cellular effects translate into sustained improvement in glucose regulation without inappropriate insulin secretion at low glucose. The involvement of TALK channels in intracellular Ca^2+^ handling provides a rationale for investigating effects beyond those achieved through K_ATP_-dependent control of membrane excitability. However, reduced hypoglycemia risk, long-term preservation of β-cell function, and additional benefit over established secretagogues remain to be demonstrated.

## Conclusion

TALK-1 and TALK-2 contribute to the regulation of β-cell Ca^2+^ handling and insulin secretion, with distinct evidence for their roles at the plasma membrane and intracellular compartments. Their effects depend on glucose concentration, metabolic stress, and interactions among islet cell types, indicating that increased insulin secretion alone is insufficient to establish improved islet function. Human genetic evidence links a rare TALK-1 gain-of-function variant to MODY and implicates variation at the *KCNK16*/*KCNK17* locus in type 2 diabetes susceptibility. Functional studies in human islets and emerging TALK-1 inhibitors provide complementary approaches to investigating the underlying mechanisms. The next challenge is to determine whether selective TALK modulation produces reproducible and sustained improvements in human islet function and whether these effects translate into improved glucose regulation *in vivo*.
